# Anthelmintic resistance in cyathostomin populations from horse yards in Italy, United Kingdom and Germany

**DOI:** 10.1186/1756-3305-2-S2-S2

**Published:** 2009-09-25

**Authors:** Donato Traversa, Georg von Samson-Himmelstjerna, Janina Demeler, Piermarino Milillo, Sandra Schürmann, Helen Barnes, Domenico Otranto, Stefania Perrucci, Antonio Frangipane di Regalbono, Paola Beraldo, Albert Boeckh, Rami Cobb

**Affiliations:** 1 Faculty of Veterinary Medicine, University of Teramo, Italy; 2 University of Veterinary Medicine, Hannover, Germany; 3 Fort Dodge Animal Health, UK; 4 Faculty of Veterinary Medicine, University of Bari, Italy; 5 Faculty of Veterinary Medicine, University of Pisa, Italy; 6 Faculty of Veterinary Medicine, University of Padua, Italy; 7 Faculty of Veterinary Medicine, University of Udine, Italy; 8 Fort Dodge Animal Health, USA

## Abstract

**Background:**

A large survey was carried out in 2008 in Europe to evaluate the efficacy of fenbendazole (FBZ), pyrantel (PYR), ivermectin (IVM) and moxidectin (MOX), i.e. the major anthelmintic molecules used in current practice against cyathostomins affecting horses. A total of 102 yards and 1704 horses was studied in three countries: 60 yards and 988 horses from Italy, 22 and 396 from the UK, 20 and 320 from Germany. The survey consisted of Faecal Egg Count Reduction Tests (FECRTs) with a faecal egg count reduction (FECR) categorization of (I) *resistance present *if FECR <90% and the lower 95% confidence limit (LCL) <90%, (II) *resistance suspected *if FECR ≥ 90% and/or LCL <90% and (III) *no resistance *if FECR ≥ 90% and LCL >90%. The calculation of FECR data was performed employing bootstrap analysis of group arithmetic means.

**Results:**

The testing of FBZ on a total of 80 yards resulted in *resistance present *on more than 80% of the UK and German yards and on significantly fewer in Italy, i.e. in 38% (p < 0.01). PYR, IVM and MOX were tested on a total of 102 yards. For PYR *resistance present *was found in 25% of the yards with no significant differences between countries. For IVM *resistance present *was encountered in one Italian and two UK yards (3%), *resistance present *to MOX was not found in any yard in any country.

**Conclusion:**

The results indicate that single and/or multiple drug resistance in equine cyathostomins is present in the three countries, is widespread particularly for FBZ and/or PYR and in one UK yard multiple resistance present was detected to FBZ, PYR and IVM. Macrocylic lactones proved to be the most effective drugs, with some evidence of resistance to IVM and highest activity of MOX, despite a single case of reduced efficacy in Germany. These data call for the development and implementation, among practitioners, owners and managers, of further plans to reduce the expansion of the anthelmintic resistant populations and to use those anthelmintics that remain effective in a manner that preserves their efficacy as long as possible.

## Background

In recent years, the spread of nematode populations resistant to parasiticides has become a serious threat for animal health, welfare and production in many areas of the world. Suppressive treatment strategies and/or abuse of anthelmintics have resulted in selection of drug-resistant parasites of horses belonging to the Cyathostominae subfamily (Nematoda, Strongylida), i.e. cyathostomins or small strongyles [1-3]. The control of horse cyathostominosis usually relies on three major classes of anthelmintics, the benzimidazoles - BZs (e.g. fenbendazole - FBZ), the tetrahydropyrimidines - THP (i.e. pyrantel-PYR salts) and the macrocyclic lactones - MLs (i.e. ivermectin-IVM and moxidectin-MOX). Resistance to BZs is widespread with prevalence up to 100% in some countries where it is now almost impossible to find susceptible parasite populations [3]. Reports in the early 90's [4] indicate that resistance to THP is presently increasing in Europe and North America as well [5]. IVM and MOX have shown full efficacy against cyathostomins until the past two years, when cases of reduced efficacy of IVM in UK [6], Germany [7] and US [8] have been published. Very recently, the failure of MLs to provide control of cyathostomins in Brazil has been also reported [9]. Indeed, such a situation represents an alarm bell ringing if one considers the important pathogenic potential played by small strongyles, as they are the cause of severe intestinal syndromes at both the adult and larval stages [9-11]. Furthermore, when the larvae encysted in the intestinal wall simultaneously emerge, they induce the potentially life-threatening "larval cyathostominosis", a colitis with loss of protein and weight, severe diarrhoea, and oedema [12-14].

The movement of horses between countries and the virtually global spread of cyathostomins, regardless of their status of susceptibility to parasiticides, [1] underline the significant need to enhance our knowledge of the actual changes in the occurrence and spread of anthelmintic resistant populations in different parts of the world. In Europe cyathostomins resistant to one ("single resistance") or more ("multiple resistance") anthelmintic class have been reported in a range of countries [15-21]. The majority of these studies have relied on a small number of horse yards in limited areas. Therefore, given the merit in geographically and numerically broader investigations of drug resistance on horse farms, the present large scale survey has evaluated the efficacy of the major drugs used in current practice against cyathostomins and the prevalence of resistant populations in three European countries.

## Methods

### Yards and animals

In 2008 a total of 146 yards and 4280 horses were screened in Europe for the presence of cyathostomin infection. These were 84 yards and 2105 horses from Italy, 32 and 1059 from the UK, and 30 and 1116 from Germany, respectively. Such a pre-treatment screening was performed with faecal egg counts (FEC) on all horses present on the yards [22]. A value of ≥ 50 eggs per gram (EPG) of faeces in 12 to 20 horses was used as a cut-off for inclusion of properties in the survey. This resulted in a total of 102 yards and 1704 horses studied in the three countries: 60 yards and 988 horses from Italy, 22 and 396 from the UK, 20 and 320 from Germany.

### Faecal Egg Count Reduction Test

All horses were subjected to a Faecal Egg Count Reduction Test (FECRT). The pre-FECRT screening prior to Day 0 was followed by random allocation of the animals in each yard to equally sized treatment groups of 4 or 5 horses each, depending on the number of horses with positive FECs. On Day 0, animals enrolled in each group were orally treated with either FBZ, PYR, IVM or MOX at the dosages recommended for the treatment of horse cyathostominosis. Each treatment was performed by veterinary practitioners for the different yards (see acknowledgments). To determine the individual pre-treatment and post-treatment EPG values, faecal samples were collected for each animal pre-dosing on Day 0 and two weeks later (Day 14). All FECs were performed at the Parasitology Laboratory at the Faculty of Veterinary Medicine, University of Teramo. Within 24 hours of receipt, the individual samples were subjected to quantitative coproscopic analysis [22].

The calculation of the FECR percentages was performed using the newly developed computer program "BootStreat" [23], based on bootstrapping methods. This program allows calculation of the mean efficacy of the treatment and provides confidence intervals based on re-sampling-bootstraps [24]. The efficacy data usually do not follow a Gaussian distribution and confidence intervals cannot be calculated. Bootstrap analysis is one approach for evaluating confidence intervals on non-Gaussian distributions. Here arithmetic means of the pre- and post-treatment FEC were used to calculate the group FECR according to the formula



and the lower and upper 95% confidence limits using a re-sampling number of 2000. Based on the methods recommended by the World Association for the Advancement of Veterinary Parasitology [22] for the detection of anthelmintic resistance in horses and ruminants, and a previous US study on large numbers of yards and horses [25], the obtained FECRs were categorized for all tested compounds as follows: (I) *resistance present *if FECR <90% and the lower 95% confidence limit (LCL) <90%, (II) *resistance suspected *if FECR ≥ 90% and/or LCL <90% and (III) *no resistance *if FECR ≥ 90% and LCL >90%. To statistically analyse differences concerning the distribution of resistance on yards in the three countries for each of the four tested compounds, the categorized results were examined using Fischer's exact test.

Post-treatment larval cultures from each yard were performed from pooled faecal samples collected from each treatment group. Pooled faeces were mixed with oak sawdust and water. Coprocultures were incubated for 10 days at 27°C and after incubation third-stage larvae (L3) were harvested using baermanization, examined using a light microscope and identified using morphological keys [26].

## Results

All four compounds were evaluated in 80 yards (50, 17 and 13 from Italy, UK, Germany respectively) where sufficient cyathostomin infected horses were available for four treatment groups. FBZ was excluded in 22 yards, where a smaller number of horses was available after EPG pre-screening (10, 5, 7 from Italy, UK, Germany respectively).

Treatment with FBZ resulted in *resistance present *in 19 out of 50 (38%), 14 out of 17 (82.4%) and 11 out of 13 (84.6%) yards, *resistance suspected *in 8 (16%), 2 (11.8%) and 0 (0%) yards and *no resistance *in 23 (46%), 1 (5.8%) and 2 (15.4%) in Italy, UK and Germany, respectively. As for PYR, *resistance present *was found in 18 out of 60 (30%), 4 out of 22 (18.2%) and 4 out of 20 (20%) yards, *resistance suspected *was found in 17 (28.3%), 2 (9.1%) and 4 (20%) yards and *no resistance *in 25 (41.7%), 16 (72.7%) and 12 (60%) for Italy, UK and Germany, respectively. Treatment with IVM resulted in *resistance present *in 1 out of 60 (1.7%), 2 out of 22 (9.1%) and 0 (0%), *resistance suspected *in 3 (5%), 1 (4.5%) and 1 (5%) and *no resistance *in 56 (93.3%), 19 (86.4) and 19 (95%) for Italy, UK and Germany, respectively. Treatment with MOX was 100% effective in all yards examined in Italy and UK. The same 100% efficacy was found for 86 out of a total of 87 horses treated with MOX in Germany, this one horse led to a FECR <100% in one German yard.

Mean percentage efficacies of different parasiticides evaluated in each enrolled yard and respective 95% confidence intervals are listed in Additional file 1 for the three countries.

Among countries, the distribution of yards showing FECR data categorized as indicative for anthelmintic resistance differed significantly only for FBZ, whereas for the other three compounds no significant differences were found. In Italy, only about one third of the farms were categorized as *resistance present *compared to more than 80% in UK and Germany (p < 0.01). On farms where all four compounds were tested, the occurrence of multiple resistance, i.e. categorization as resistance present for more than one compound, was seen on 10 (20%, n = 50) Italian, 5 (29.4%, n = 17) UK and 3 (23.1%, n = 13) German farms. Multiple resistance always included FBZ and PYR, except for one Italian (FBZ and IVM) and two UK (FBZ and IVM, and FBZ and PYR and suspected for IVM) yards. In one of the UK farms resistance was found to FBZ, PYR and IVM, thus encompassing all the three chemical drug classes. MOX retained 100% efficacy on this site.

Evaluation of results for individual horses, showed 0-100% range of efficacy for FBZ and PYR in each of the three countries (Table 1). IVM showed a range of 0-100% efficacy in UK and Italy, with incomplete efficacy found in four Italian, three UK and one German yard (Table 1). MOX was 100% effective in all treated horses from the three countries with the exception of a single horse in a yard from Germany, which displayed a FECR <100% for this compound (Additional file 1 and Table 1). Additional File 1 shows mean percentages of FECR and respective confidence intervals, set between 0 and 100%.

**Table 1:** Minimum (min) and maximum (max) percentage (%) values of faecal egg count reduction after treatment with fenbendazole (FBZ), pyrantel (PYR), ivermectin (IVM) and moxidectin (MOX) evaluated against cyathostomins in yards (Y) and horses (H) located in Italy, UK and Germany.

	**Italy % (min-max)**		**UK % (min-max)**		**Germany % (min-max)**	
			
	**Y**	**H**	**Y**	**H**	**Y**	**H**
FBZ	32.4-100	0-100	15-100	0-100	11.5-100	0-100
PYR	50.7-100	0-100	70.5-100	0-100	75-100	0-100
IVM	80-100	0-100	70-100	0-100	95.7-100	78.5-100
MOX	100	100	100	100	95-100	75-100

**Figure 1:**
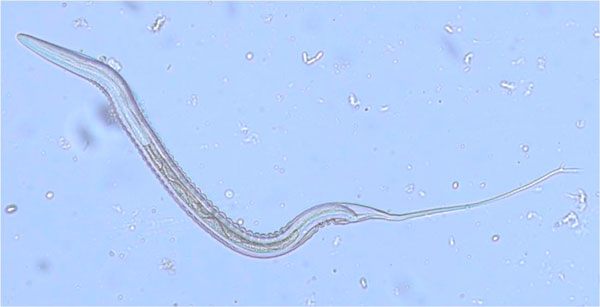
**Post-treatment larval culture: cyathostomin third stage larva**.

The microscopic examination of the *in vitro *L3s collected by the Baermann technique performed on the cultured post-treatment faecal pools according to their respective midgut cell numbers showed that they belonged exclusively to the Cyathostominae subfamily (Figure 1).

## Discussion and conclusion

This multinational survey, the largest carried out so far to evaluate the efficacy of major anthelmintic drugs against equine cyathostomins, demonstrated that resistant cyathostomin populations to FBZ and PYR are widespread in Europe, with higher prevalence for the former drug. Individual horse FECs showed considerable variability in the susceptibility status to FBZ, PYR and even IVM within yards (Table 1).

The calculation of the 95% confidence interval provided strong evidence for the significance of the FECR% and for the presence of drug resistance in cyathostomins affecting horses in Italy, UK and Germany. A single-dose use of FBZ was ineffective in all but 3 yards examined in the UK and demonstrated reduced efficacy in almost all German properties. In Italy, the efficacy of FBZ was found to be reduced in about one third of the yards. The data generated in this survey suggest that PYR has also lost efficacy against small strongyles in the European countries investigated. Indeed, *reduced efficacy *to PYR was found in all three countries involved in this survey and *resistance present *in about 20-30% of the examined yards. However it should be taken into consideration that the intrinsic efficacy of PYR appears to be generally lower than that of the ML drugs [27].

This survey confirmed that the efficacy of MLs against cyathostomins remains high. However, there were yards (one in Italy and two in the UK) where the FECR efficacy of IVM was observed to be <90% (LCL<90%) which is considered as resistance. While a reduced efficacy of IVM has been already detected in UK in the past years [6], the present results report the first evidence for reduced efficacy of this molecule in controlling small strongyles in Italy as well.

The results of this survey indicate that MOX is 100% effective in treating the infection caused by cyathostomins in Europe, the only exception was a single horse in yard n. 19 from Germany (Additional file 1), which was positive for a post-treatment FEC of 50 (i.e. 75% FECR) two weeks after the treatment (Table 1). Such a result led to a FECR of <100% for MOX in this sole yard out of the 102 yards examined in this survey. Noteworthy, MOX was recently reported to no longer provide control of cyathostomin infection in Brazil [9].

Previous studies have already shown that cyathostomin populations resistant to BZs are present in Europe. The prevalence of resistance in Switzerland was shown to be greater than 50% of yards examined [28], and prevalence rates up to 75-100% were reported in Sweden [29], Denmark [15], England [30], Slovak Republic [31] and Germany [18]. Similarly, resistance to PYR has been previously described both in Europe [4,19,20,32] and the USA [25,33]. Therefore, the overall outlook demonstrated by this broad survey undoubtedly enhances the concern regarding the spread of anthelmintic resistance in horse cyathostomins. This information provides some basic knowledge to prompt and improve awareness of resistance and to stimulate the use of parasiticides in programmes that minimize selection for resistance. In fact, erroneous practices such as underdosing, over-use, and off label use of anthelmintic products are known to be the basis for the selection of resistant parasite populations. The results reported here highlight that present strategies for worm control in horses have to be re-considered. For instance, the low prevalence of resistance to THP found in most of the world with the exception of the US, was explained in the past by the common practice of daily feeding of low-dose PYR tartrate in North America. This might have a strong impact in the selection for resistance also to other PYR salts [25]. The levels of reduced efficacy of PYR found in all three EU countries in this survey, where the daily feeding is not practiced, demonstrate that such a programme is just one of many practices leading to selection for drug-resistant parasites. Another relevant example is represented by the widespread loss of efficacy of BZs and PYR in UK, where the over-treatment of thoroughbreds in the past likely caused the spread of worm populations resistant to these classes [34]. Worthy of note is that recently Dudeney et al. [35] have reported that resistance to MLs is starting to develop in cyathostomins in the UK. Of particular importance and concern is the finding of relatively high proportions of yards exhibiting signs of resistance to more than one drug class in parallel and most noteworthy the first finding of multiple FBZ/PYR/IVM resistance cyathostomins in UK.

Some drug classes, such as the cyclooctadepsipeptides, paraherquamides, aminoacetonitrile derivatives, are the novel anthelmintics that are being used [36-38] but their potential applicability to horse cyathostomins is unknown. Therefore, if the extent of resistance to MLs increases and spreads, horse owners and equine veterinary practitioners will face an important problem with no ready solution. Given the real threat that the resistance to MLs may worsen or spread through horse movement, there is an urgent need to appropriately use the remaining effective drugs, especially MOX, in order to preserve their efficacy.

Horse yards should be regularly monitored not only for resistance to BZs and PYR, but also to MLs. Over-use of IVM and MOX could be avoided in those properties where the other anthelmintics are still highly effective. Specifically, the drug resistance status should be established on each property at least on an annual basis and, where effective, BZs and PYR should be also administered to reduce the pressure on MLs [9,25]. It has been suggested that each newly introduced horse should be quarantined and treated with IVM, since the still constant 100% efficacy of MOX could hide the first signs of resistance to IVM [9]. However, in light of the findings in this study, the use of IVM as a quarantine treatment may allow the unintended introduction of resistant cyathostomins. An alternative would be to use MOX as the best option for avoiding the introduction of resistant parasites to the site. The active monitoring of anthelmintic effectiveness needs to take into consideration the egg reappearance period of IVM [7]. If the IVM-treated animal sheds cyathostomin eggs after 4 weeks rather than 6 to 8 weeks, the status of the animal is worth further evaluation with sensitive quantitative copromicroscopic evaluations [9].

Another key point to be taken into account is the maintenance of the *refugia*, which provide an useful dilution of the resistant genes in the parasitic populations. The preservation of the *refugia *while controlling cyathostomins at the same time requires routine FECs to identify the horses actually needing an anthelmintic treatment. This approach is different from the commonly used interval dose program (i.e. treat-all-animals) and would avoid the treatment of animals that are currently being treated more frequently than necessary. Usually, the majority of the horses on a property shows a low level of faecal egg shedding, while the greater part of the cyathostomin populations is present in a small proportion of horses that shows moderate-high FECs [5,25,39]. Thus a knowledge of the FEC values is recommended as the cornerstone for the necessity to treat. An indicative minimum cut-off of 200 EPG [9,25,39] has been recommended as a guide to the need for treatment of individual horses. This would achieve the dual goal of controlling cyathostomin-induced health problems while simultaneously reducing pasture contamination. The analysis of the screening FECs data pre-FECRT (not shown) of the present survey demonstrated that there was a higher percentage of "high egg shedders" (i.e. >150 EPG) than "low or null egg shedders" (i.e. <150 EPG), thus confirming that current control programs are not ideal and providing further support for the value of conducting FECs before planning any anthelmintic treatment in a yard. Leaving a proportion of horses untreated would maximize the *refugia *with a little impact on overall control, as horses with low egg count are not important sources of environmental contamination. Additionally, the egg shedding from untreated animals would dilute the presence on the pasture of any eggs shed by treated animals possibly infected with resistant populations. In this way the selection pressure would be progressively reduced [9,25].

In conclusion, given the strong impact that resistant cyathostomins can and will likely have on horse health, future parasite control plans should be based on integrated measures represented by correct use of anthelmintics and other approaches, such as adequate pasture hygiene, low stocking rates and mixed grazing with other animals [9,40,41]. It is essential that owners, managers and veterinary practitioners take an active and leading role in planning and monitoring effective and appropriate worm control programs for horses.

## Competing interests

Fort Dodge Animal Health provided financial and logistic support to this study. The authors declare that there are no competing interests and that the conceptual design, the conduct, the interpretation or any other scientific aspect of the study have not been influenced by the FDAH support.

## Authors' contributions

DT: Contributed to the design of the study, was responsible for the Italian farms study and for the copromicroscopical analysis of all faecal samples from each of the Countries and drafted the manuscript; GvSH: Contributed to the design of the study, was responsible for the German farms study, participated in the statistical analysis and in drafting the manuscript; JD: Participated in the faecal sampling for the German farms and in the statistical analysis; PM: Participated in the faecal sampling for the Italian farms, coordinated and conducted the copromicroscopical analysis; SS: Participated in the faecal sampling for the German farms; HB: Contributed to the design of the study, was responsible for the UK farms study; DO: Participated in yards enrolment in Italy and faecal sampling; SP: Participated in yards enrolment in Italy and faecal sampling; AFdR: Participated in yards enrolment in Italy and faecal sampling; PB: Participated in yards enrolment in Italy and faecal sampling; AB: Initiated the study and contributed to the design of the study; RC: Initiated the study and contributed to the design of the study

## Supplementary Material

Additional file 1 Table showing mean percentages of faecal egg count reduction after treatment with fenbendazole (FBZ), pyrantel (PYR), ivermectin (IVM) and moxidectin (MOX) evaluated against cyathostomins in a total of 102 horse yards located in Germany, Italy and UK. The faecal egg count reductions (%) and respective 95% confidence intervals (CI) (set between 0 and 100%) were calculated using the Bootstreat programme using the formula FECR = 100 * (1 - arithmetic mean of FEC post treatment/arithmetic mean of FEC per treatment) with 2000 repeats. H: treated horses in each yard; G: number of horses in each treatment group. Click here for file
