# Supplementary material for: Anthelmintic resistance in cyathostomin populations from horse yards in Italy, United Kingdom and Germany
Source: Parasit Vectors. 2009 Sep 25;2(Suppl 2):S2. doi: 10.1186/1756-3305-2-S2-S2 (PMC2751838; doi:10.1186/1756-3305-2-S2-S2)
Supplement: Additional file 1 — Table showing mean percentages of faecal egg count reduction after treatment with fenbendazole (FBZ), pyrantel (PYR), ivermectin (IVM) and moxidectin (MOX) evaluated against cyathostomins in a total of 102 horse yards located in Germany, Italy and UK. The faecal egg count reductions (%) and respective 95% confidence intervals (CI) (set between 0 and 100%) were calculated using the Bootstreat programme using the formula FECR = 100 * (1 - arithmetic mean of FEC post treatment/arithmetic mean of FEC per treatment) with 2000 repeats. H: treated horses in each yard; G: number of horses in each treatment group. [file 1756-3305-2-S2-S2-S1.pdf]

Additional file 1

Mean percentages of faecal egg count reduction after treatment with fenbendazole (FBZ), pyrantel pamoate (PYR), ivermectin (IVM) and moxidectin (MOX) evaluated against cyathostomins in a total of 102 horse yards located in Germany, Italy and UK. The faecal egg count reductions (%) and respective 95% confidence intervals (CI) (set between 0 and 100%) were calculated using the Bootstreat programme using the formula FECR= 100 * (1 − arithmetic mean of FEC post treatment/arithmetic mean of FEC per treatment) with 2000 repeats. H: treated horses in each yard; G: number of horses in each treatment group**.**

Red Values: *resistance present* (FECR <90% and LCL <90%)
Blue values: *resistance suspected* (FECR ≥90% and/or LCL <90%)
Black values: *no resistance* (FECR ≥90% and LCL >90%)

Country	Yard ID	H/G	FBZ	95% CI	PYR	95% CI	IVM	95% CI	MOX	95% CI
Germany	1	20/5	79	23-100	94	76-100	100		100	
	3	20/5	28	0-93	90	51-100	93	75-100	100	
	4	20/5	0	0-51	100		100		100	
	5	12/4			100		100		100	
	7	16/4	46	44-100	83	44-100	100		100	
	8	16/4	0	0-53	100		100		100	
	9	16/4	31	0-100	100		100		100	
	10	16/4	100		92	43-100	100		100	
	11	12/4			83	42-100	100		100	
	13	20/5	25	0-97	100		100		100	
	14	16/4	43	0-76	100		100		100	
	15	20/5	89	72-100	84	64-99	100		100	
	16	16/4	100		100		100		100	
	18	12/4			100		100		100	
	19	20/5	30	0-100	100		100		97	88-100
	21	20/5	44	0-90	64	0-100	100		100	
	23	12/4			100		100		100	
	24	12/4			100		100		100	
	27	12/4			95	70-100	100		100	
	30	12/4			100		100		100	
Italy	B1	12/4			91	41-100	100		100	
	B2	16/4	100		100		100		100	
	B3	16/4	95	80-100	100		100		100	
	B4	12/4			98	92-100	100		100	
	B5	16/4	100		95	72-100	100		100	
	B6	16/4	100		100		100		100	
	P1	20/5	71	16-100	63	2-89	100		100	
	P2	16/4	100		91	82-100	100		100	
	P3	16/4	100		92	73-100	100		100	
	P4	20/5	97	90-100	63	3-100	100		100	

Country	Yard ID	H/G	FBZ	95% CI	PYR	95% CI	IVM	95% CI	MOX	95% CI
Italy	P5	20/5	98	92-100	96	90-100	97	92-100	100	
	P6	12/4			97	88-100	100		100	
	P7	16/4	100		100		100		100	
	P8	16/4	100		100		100		100	
	C1	20/5	77	0-100	100		100		100	
	C2	12/4			100		100		100	
	C3	16/4	100		100		100		100	
	C4	20/5	96	84-100	100		100		100	
	C5	20/5	91	77-100	89	56-100	96	76-100	100	
	C6	20/5	96	88-100	97	89-100	100		100	
	L1	20/5	41	0-89	44	0-81	100		100	
	L2	20/5	61	0-96	100		100		100	
	L3	20/5	63	0-95	92	81-99	95	73-100	100	
	L4	20/5	3	0-86	100		100		100	
	L5	20/5	0	0-64	90	73-100	100		100	
	L6	20/5	69	33-100	100		100		100	
	L7	16/4	94	79-100	100		100		100	
	L8	16/4	100		100		100		100	
	L9	12/4			100		100		100	
	A1	20/5	83	34-100	100		46	0-100	100	
	A2	16/4	100		100		100		100	
	A3	16/4	93	67-100	91	69-100	100		100	
	A4	16/4	100		95	84-100	100		100	
	A5	16/4	100		96	86-100	100		100	
	A6	16/4	98	92-100	97	91-100	100		100	
	A7	16/4	100		97	89-100	100		100	
	A8	16/4	86	48-100	63	0-100	100		100	
	A9	16/4	100		37	0-96	100		100	
	U1	16/4	100		100		100		100	
	U2	12/4			100		100		100	
	F1	20/5	56	0-99	60	0-93	100		100	
	F2	20/5	74	0-100	100		100		100	
	F3	20/5	48	0-94	71	31-100	100		100	
	F4	16/4	77	59-91	83	64-95	100		100	
	F5	16/4	69	0-96	87	76-98	100		100	
	V1	16/4	83	44-100	97	84-100	100		100	
	V2	12/4			95	52-100	94	68-100	100	
	V3	12/4			76	15-100	100		100	
	V4	12/4			88	12-100	100		100	
	V5	12/4			90	71-100	100		100	
	T1	16/4	32	0-91	38	0-100	100		100	
	T2	16/4	86	41-100	83	46-100	100		100	
	T3	16/4	100		98	94-100	100		100	
	T4	16/4	97	73-100	70	0-100	100		100	
	T5	16/4	100		78	54-100	100		100	
	T6	16/4	99	93-100	100		100		100	
	T7	16/4	89	54-100	90	60-100	100		100	

Country	Yard ID	H/G	FBZ	95% CI	PYR	95% CI	IVM	95% CI	MOX	95% CI
**Italy**	T8	16/4	91	79-100	93	79-100	100		100	
	T9	16/4	98	93-100	87	41-100	100		100	
	T10	16/4	100		79	9-100	100		100	
**UK**	2	20/5	0	0-80	74	41-93	97	88-100	100	
	3	16/4	0	0-48	85	65-100	100		100	
	5	20/5	0	0-44	88	51-100	89	56-100	100	
	6	20/5	0	0-72	100		100		100	
	7	12/4			100		100		100	
	9	20/5	80	42-98	97	85-100	100		100	
	10	20/5	96	88-100	100		100		100	
	11	20/5	27	0-92	100		100		100	
	12	12/4			100		100		100	
	15	20/5	0	0-73	63	3-100	100		100	
	16	12/4			100		100		100	
	26	12/4			100		100		100	
	31	20/5	68	27-90	94	71-100	100		100	
	32	12/4			100		100		100	
	33	20/5	0	0-71	100		72	30-100	100	
	34	20/5	80	1-100	100		100		100	
	35	20/5	100		100		100		100	
	36	20/5	30	0-78	100		100		100	
	37	20/5	94	63-100	100		100		100	
	39	20/5	28	0-97	100		100		100	
	40	20/5	66	10-98	100		100		100	
	41	20/5	54	0-100	100		100		100	
